# Targeting the PD-1/PD-L1 axis for cancer treatment: a review on nanotechnology

**DOI:** 10.1098/rsos.211991

**Published:** 2022-04-13

**Authors:** Tuan Hiep Tran, Thi Thu Phuong Tran

**Affiliations:** ^1^ Faculty of Pharmacy, PHENIKAA University, Hanoi 12116, Vietnam; ^2^ Department of Life Sciences, University of Science and Technology of Hanoi Vietnam Academy of Science and Technology, Hanoi, Vietnam

**Keywords:** PD-1/PD-L1, nanoparticles, immunotherapy, cancer treatment, combination therapy

## Abstract

Although nanomedicines have been in the oncology field for almost three decades with the introduction of doxil, only a few nanomedicine products have reached approval. Can nanotechnology be a realistic tool to reduce the number of hospital beds? At present, several clinically approved anti-PD-1/PD-L1 antibodies or CAR T cell-based therapies are available; however, the immunotherapy field is far from mature. Will immunotherapy be the fourth pillar of cancer treatment? In this review, we summarized the current status of immunotherapy using PD-1/PD-L1-targeting nanocarriers. The knowledge on material science, therapeutic agents and formulation designs could pave the way for high-efficacy treatment outcomes.

## 1. Introduction

Cancer management has been progressing owing to the developments in diagnosis and treatment. However, to reach a cancer cure, especially in the cases of late-stage cancers, more advanced technologies and concerted effort are required. In combination with standard treatments, cancer immunotherapy has shown huge potential in improving treatment outcomes [1]. Briefly, cancer immunotherapy includes the use of monoclonal antibodies, cytokines, proteins, small molecules and cells, to manipulate the immune system by which the body can use its own defence mechanisms against the development of cancer [2]. Despite encouraging clinical outcomes, significant challenges still remain, including the ‘cold’ tumour microenvironment (TME), T-cell exhaustion and immune escape that could collectively attenuate the efficacy of immunotherapy [3].

Under normal conditions, immune cells recognize and eliminate cancer cells as foreign pathogens. Unfortunately, tumours are capable of suppressing cellular signalling and metabolism via enhanced immune receptor inhibition on the tumour surface or elevated signalling inhibition among immune cells [4]. Immune checkpoint blockade therapies especially those targeting the programmed cell death protein-1 receptor (PD-1) and PD-ligand-1 (PD-L1) axis have shown significant clinical efficacy [5]. To further improve this therapy, scientists are now using nanotechnology-based medical treatments, devices and instruments to increase efficacy, safety, sensitivity and personalization [1,6,7].

In this review, we summarized the approaches and designs targeting the PD-1/PD-L1 axis using nanotechnology for cancer treatment. This overview is premised on the biological fundamentals of PD-1/PD-L1 in cancer treatment and the principles of nanotechnology, including the properties of nanoparticles (NPs). Active agents such as antibodies, peptides, siRNAs, miRNAs and small molecules have been routinely incorporated into various nanosystems for therapeutic, targeting or diagnostic purposes. Although nanomedicine in anti-cancer therapy employs active agents in mono-therapeutic or combinatorial cancer regimens, the exact mechanism of action of targeted therapies remains poorly understood. Therefore, by weighing on the pros and cons of targeted immunotherapy, herewith, we suggest a potential prospective of the development of PD-1/PD-L1-targeting immunotherapy.

## 2. PD-1/PD-L1 axis: a potential therapeutic target

A tumour is a complex and heterogeneous tissue area which is composed of cancer cells, stromal cells, vascular networks and many other cellular and non-cellular components. This network creates a TME which challenges the therapies. The immune system which is expected as the first reaction force is also divided into ‘hot’ and ‘cold’ attributed to the infiltration of immune cells and proinflammatory cytokines [8]. ‘Cold’ tumours tend to be surrounded by cells that are able to suppress the immune response and keep T cells from attacking the tumour cells and killing them. Of note, prognosis and prediction of therapy are closely associated with the tumour-infiltrating immune cells [9].

Immune checkpoint blockade therapies are FDA-approved treatment regimens for various types of cancer in which therapeutic agents are used to restore the function of immune cells, primarily effector T cells [10]. However, within the complex immune system landscape, the immune checkpoint molecules such as cytotoxic T lymphocyte antigen 4, PD-1 and PD-L1 are key molecules contributing to the homeostasis of physiological biology. For example, PD-1 is an immune modulator that maintains peripheral tolerance and T-cell responses under normal conditions. The engagement of PD-1 and PD-L1 was found to interfere the T-cell receptor (TCR) signal transduction and CD28-co-stimulation. In detail, engaged PD-1 recruits SHP1 and SHP2 phosphatases to its tyrosine-phosphorylated ITIM and ITSM motifs leading to the termination of ZAP70 and PI3 K phosphorylation—the key signalling of TCR signal transduction and CD28-co-stimulation, respectively [11]. Thus, the activation of the PD-1 signalling axis leads to decreased local T-cell responses and reduced tissue damage [12].

In the context of effector lymphocytes, cancer cells are foreign pathogens that need to be eliminated. However, the dynamic and complex cellular networks within the TME could enhance the development of tumour resistance mechanisms, such as overexpressing PD-L1, a counterpart of PD-1, which enable cancer cells to escape immune elimination. PD-1 is constitutively expressed on exhausted T cells, B cells, monocytes, dendritic cells, regulatory T cells and natural killer (NK) T cells, whereas PD-L1 is upregulated in tumour cells, both in solid and haematologic malignancies [5].

To enhance the knowledge on this immune checkpoint, many research groups have developed the PD-1/PD-L1 inhibitors to enhance T-cell infiltration to the TME, activate intratumoral cytotoxic T lymphocytes and reduce the number of immunosuppressive Tregs. Remission of tumour growth has been achieved via this treatment strategy [10], indicating the promising potentials of this ‘normalization’ strategy for immune checkpoint blockade therapies. Together with the antibodies, the ability of siRNAs, miRNAs, peptides and other immune small molecules to interrupt the interaction between PD-1 and PD-L1 has already been validated [13]. Therefore, the potential use of these active agents offers a new window of hope in cancer treatments.

## 3. Nanoparticles: a novel concept for enhanced cancer treatment efficacy

**Figure 1.**
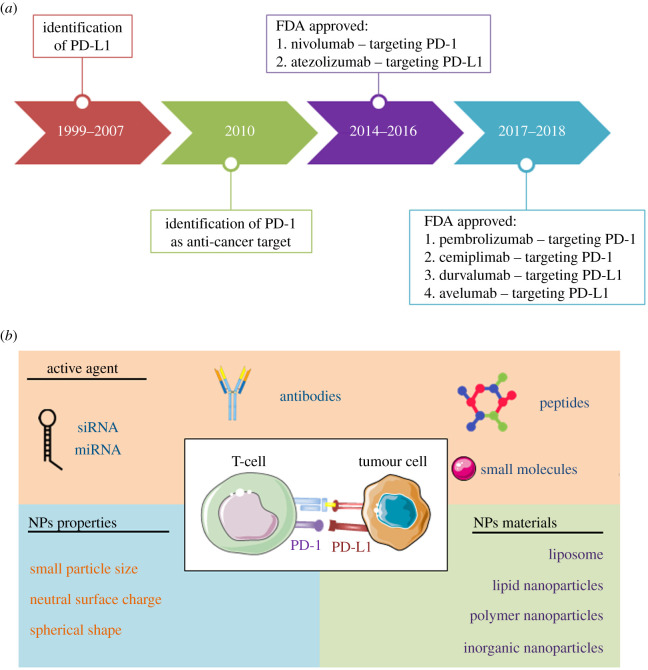
PD-1/PD-L1-targeted anti-cancer therapies. (*a*) Timeline of the development of PD-1/PD-L1 as anti-cancer target and (*b*) the components of nanosystems for PD-1/PD-L1-targeted therapies.

To achieve maximum treatment benefit, the types of NPs used should meet several criteria (figure 1). Particle size is possibly the first and most important factor that impacts their distribution in the body and immunogenicity. A size of approximately 100 nm is considered a suitable range for the NPs to be trafficked to lymph nodes or accumulated into tumour sites [14]. Additionally, although the cylindrical NPs might induce a stronger immune response compared with other types, the spherical particles are easier to fabricate, resulting in their greater popularity.

NPs with positive surface charge can induce a higher immunogenicity compared with those of neutral or negatively charged NP formulations, and neutral NPs have lower toxicity. Therefore, a charge between −10 and +10 mV is considered a suitable range for the design of NPs [15]. Notably, the addition of ligands could enhance the target specificity of active agents toward tumours, whereas PEG-conjugated NPs could prolong the bioavailability of therapeutic agents with prolonged blood circulation, thereby potentiating the application of NPs in anti-cancer therapy [16]. Overall, it should be an optimization process, case by case, to obtain a suitable nanocarrier for cancer immunotherapies.

### 3.1. Anti-PD-1/PD-L1 inhibitor-bearing nanocarriers for anti-cancer therapies

#### 3.1.1. Antibodies

**Figure 2.**
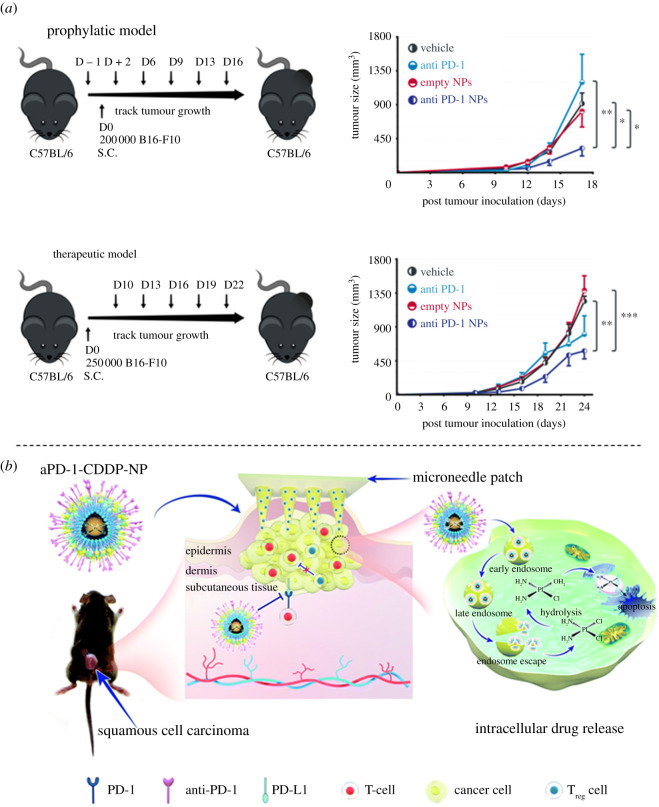
Development of anti-PD-1 antibodies-based nanosystems for cancer immunotherapy. (*a*) Photothermal therapy mediated by phase-transformation NPs facilitates the delivery of anti-PD1 antibody and synergizes with anti-tumour immunotherapy for melanoma [18] (licensed under a Creative Commons Attribution). (*b*) Micro-needles loaded with anti-PD-1–cisplatin NPs for synergistic cancer immuno-chemotherapy [19] (licensed under a Creative Commons Attribution).

The immune checkpoint inhibitor anti-PD-1 antibody has clinical potential for the treatment of cancer. In the context of NP development, anti-PD-1 antibody could be conjugated onto the surface of NPs to interrupt the PD-1/PD-L1 axis or encapsulated into nanocarriers which are then released and activated at the tumour site. Zhang *et al.* employed cargo-free NPs combined with an anti-PD-1 antibody to treat metastatic breast cancer. They administered these nanosystems to 4T1-bearing mice and observed a change in local immune suppression that significantly slowed tumour growth and resulted in a survival benefit [17]. In an earlier study, an anti-PD-1 antibody was encapsulated into PLGA NPs that were transported to the spleen, which led to an increased activation of the immune system via elevated proliferation of T cells and secretion of cytokines. Consequently, these nanosystems resulted in the suppression of murine melanoma tumour growth, therapeutically and prophylactically (figure 2*a*) [18].

In addition to encapsulating anti-PD-1 antibody to PLGA NPs, Zhang *et al.* integrated iron oxide NPs and perfluoropentane that were released under laser exposure. Under the exposure of 660 nm laser, the encapsulated iron oxide NPs could absorb the energy then generate heat that could eradicate the cancer cells as the photothermal therapy. After systemic administration, these nanosystems accumulated at the tumour site without toxicity to the mice and led to enhanced infiltration of CD8^+^ T cells. This combination of photothermal therapy and antibody-mediated immune checkpoint blockade immunotherapy effectively suppressed tumour growth [20].

Using a similar strategy of combining photothermal therapy and immunotherapy, Cao *et al.* used copper sulfide NPs as the near-infrared light-absorbing agent whereas the Toll-like receptor 9 agonist-CpG was co-delivered that improved the median survival time of mice re-inoculated with ID8-ip1-Luc tumour cells at a distant site (peritoneum). The observed synergistic effect in this study suggested the potential of combination therapy [21].

To enhance the efficacy of chemotherapy, some research groups have developed various types of nanosystems. Lan *et al.* incubated anti-PD1 antibody with lipid-coated cisplatin NPs to form anti-PD-1–cisplatin NPs. This nanosystem was further applied to a micro-needle system that activated the immune system to inhibit tumour growth. Moreover, this nanosystem could increase the infiltration of CD4^+^ T and CD8^+^ T cells into tumour tissues and decrease CD4+FOXP3+ Tregs relative to the total number of CD4^+^ tumour-infiltrating lymphocytes (figure 2*b*) [19]. In another study, Geng *et al.* combined engineered monoclonal anti-PD-1 antibodies with Mn^2+^-drugs to form multi-functional NPs. Red blood cell membranes were used to coat on the surface of nanosystems which improve the dispersity and stability of NPs, whereas the loading of doxorubicin and the photosensitizer chlorin e6 served the purposes of chemotherapy and phototherapy, respectively. Strikingly, this combination of chemotherapy and photodynamic therapy not only induced a significant decrease in the primary tumours, but also restricted the growth of distant tumours [22].

In regard to radiation therapy, Chen *et al*. developed cisplatin-loaded poly(L-glutamic acid)-graft-methoxy poly(ethylene glycol) complex NPs plus anti-PD1. The administration of this nanosystem increased the infiltration CD8^+^ T cells and secretion of chemokine (C–X–C motif) ligand 10 (CXCL10). Tumour growth was significantly suppressed via the synergistic effect of radiotherapy, chemotherapy and immunotherapy [23].

#### 3.1.2. siRNAs

Programmed cell death ligand 1 (PD-L1) small-interfering RNAs (siRNAs) are widely used to silence the expression of PD-L1 on the surface of tumour cells. This downregulation could strengthen the activity of T cells, resulting in the attenuation of tumour growth. In a pioneer approach, PD-L1 siRNA was loaded into folic acid (FA)-functionalized polyethylenimine (PEI) polymers, by which the targeting moiety improved the uptake of siRNA into SKOV-3-Luc epithelial ovarian cancer cells. About half of PD-L1 was knocked down and the sensitivity of T cells was increased twofold [24]. In a subsequent study, PD-L1 siRNA was encapsulated into human serum albumin (HSA) (plasmid/stPEI/HSA) NPs. These NPs could be easily internalized and had low cytotoxic effect, leading to a reduction in the expression of PD-L1 by 21.95% in CT26—a mouse colon carcinoma cell line [25].

The development of phototherapy could pave the way for its combinatorial application with immunotherapy. Liu *et al.* created gold nanoprisms to carry PD-L1 siRNA (GNP-siRNA). The GNPs-siRNA suppressed the expression of PD-L1 in HCC827 cells. Moreover, laser irradiation at GNPs generates heat for thermal ablation of tumour, which further augments the synergistic effect of GNPs-siRNA phototherapy in cancer treatment [26].

Li *et al.* applied dual-blockade immune checkpoints, including PD-L1 siRNA and indoleamine 2,3-dioxygenase (IDO) inhibitor, for breast cancer treatment. Both agents were loaded into a self-assembled peptide system, which could recruit and penetrate tumour-homing peptides, thereby, significantly inhibited the expression of PD-L1 in 4T1 cells. Moreover, the IDO inhibitor reduces the production of kynurenine—a metabolic agent that impairs the function of activating T cells. Following the administration of this nanosytem, the tumour growth was found to be decreased along with the increase of infiltrated CD4^+^ and CD8^+^ T cells and of level of IFN-γ and IL-2 in isolated tumour tissue. This result suggests the potential of carrier in cancer treatment exploiting immunotherapy [27].

For theranostic purposes, Luo *et al.* loaded PD-L1 siRNA into FA-functionalized PEI superparamagnetic iron oxide NPs. This target-specific nanocarrier with a size of 120 nm facilitated the application of magnetic resonance imaging (MRI) for tumour diagnosis, and the downregulation of PD-L1 resulted in the enhanced secretion of cytokines toward effector T-cell proliferation for gastric cancer treatment [28]. In another study, Pacheo-Torres *et al.* developed NPs containing a dextran scaffold, PD-L1 siRNA and Cy 5.5 NIR probe, which effectively inhibited the expression of PD-L1 in MDA-MB-231 cells. Therefore, theranostic nanosystem allowed a concurrent diagnosis and anti-cancer therapy via an innovative siRNA delivery approach [29].

**Figure 3.**
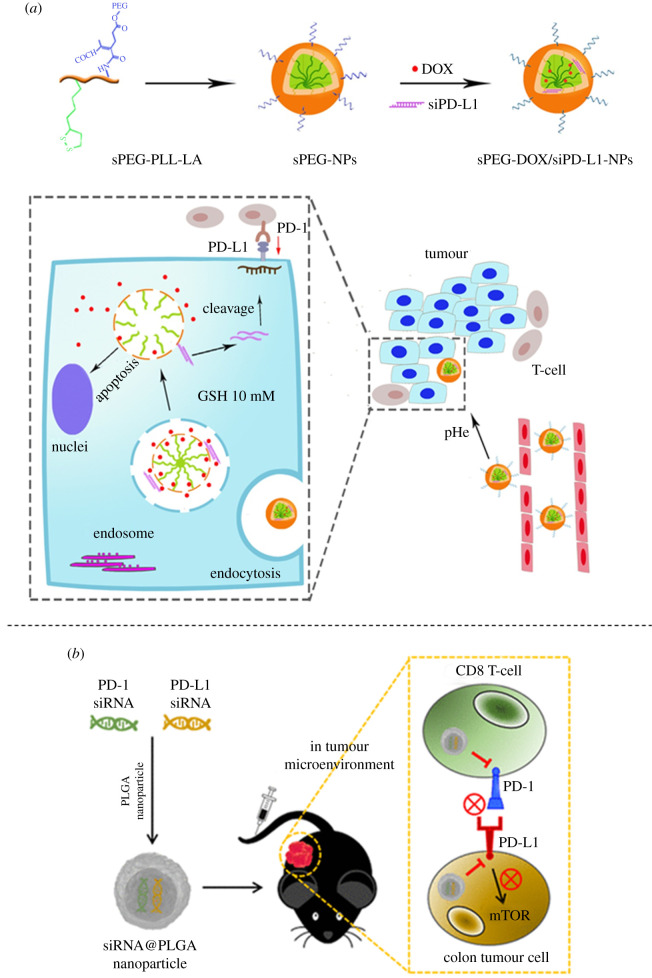
Development of siRNAs-based nanosystems for immunotherapy. (*a*) Stimuli-responsive NPs for the co-delivery of chemotherapeutic agents doxorubicin and siPD-L1 to enhance the anti-tumour effect. Reprinted with permission from [32]. (*b*) PLGA NPs co-delivering siRNAs against programmed cell death protein-1 and its ligand gene for suppression of colon tumour growth. Reprinted with permission from [33]. Copyright 2021 American Chemical Society.

Cell membranes coating could favour the delivery of NPs by the compatibility with host body. Chen *et al.* used the cancer cell membrane to cover the PLGA NPs containing doxorubicin and PD-L1 siRNA. This strategy enhanced the uptake of nanosystem into the cancer cells leading to the significant downregulation of PD-L1 and the death of cancer cells [30]. With a similar active combination, doxorubicin and PD-L1 siRNA, Mu *et al.* recruited stem cell membrane-coated polydopamine NPs (PDA–DOX/siPD-L1@SCM) as the delivery carrier. PDA–DOX/siPD-L1@SCM performed a great efficacy in PCa bone metastases by the synergistic effect [31]. In another work, doxorubicin and PD-L1 siRNA were loaded into a stimuli-responsive polymer with a poly-L-lysine–lipoic acid reduction-sensitive core and a tumour extracellular pH-stimulated shedding polyethylene glycol layer. This NP accumulated selectively to tumour site, downregulated the PD-L1 expression, increased the CD8^+^ T cells population in the tumour and reduced the tumour size (figure 3*a*) [32]. Toward the treatment using chemo-immunotherapy, Wan *et al.* co-delivered siRNA-PD-L1 and doxorubicin in a reactive oxygen species responsive NP, modified with the HAIYPRH peptide that targets to transferrin receptor. The enhancement of tumour uptake and ROS response triggered a burst release of DOX leading to cell apoptosis, PD-L1 suppression and tumour volume reduction [34]. The chemo-immunotherapy does not just stay at doxorubicin. Imatinib (IMT) was also recruited in a liposomal NP with PD-L1 siRNA. This co-delivery was more advanced than the plain cocktail of siRNA+IMT by almost doubling the cancer cell apoptosis (60% versus 35%), fourfolding the IFN-γ generation in spleen [35]. In the other study, beside targeting PD-L1, Wang *et al.* tried to inhibit TGF-β receptor using LY2157299. The TGF-β receptor inhibitor and PD-L1 siRNA were carried on and significantly increased the infiltration of CD8^+^ T cells to tumour and triggered anti-tumour immunity to synergistically suppress tumour growth. The efficiency of this delivery system was evaluated in both a subcutaneous Panc02 xenograft model and an orthotopic tumour model [36].

PD-L1 siRNA can be used in combination with other siRNAs. Bastaki *et al.* co-delivered STAT3 and PD-L1 siRNAs into hyaluronate-TAT trimethyl/thiolated chitosan NPs. In melanoma and breast cancer, the expression of genes encoding PD-L1 and oncogenic transcription factor STAT3 were suppressed, leading to the inhibition of angiogenesis, proliferation, migration and tumour growth *in vivo* [37]. Likewise, Kwak *et al.* used both siRNAs of PD-1 and PD-L1 in a PLGA NP to promote tumour-specific effector T cells. It was found that this co-delivery showed a great efficacy compared with mono-therapeutic agent delivery. Co-silencing of PD-1 and PD-L1 effectively suppressed tumour growth and prolonged tumour inhibition in colon cancer (figure 3*b*) [33].

PD-L1-siRNA can also be combined with immune-stimulatory IL-2 encoding plasmid DNA in PEI-lipid NPs. PD-L1-siRNA/IL-2 DNA-PEI-LNPs downregulated PD-L1 in A549 cancer cells and increased IFN-γ and TNF-α levels [38]. Wu *et al.* combined PD-L1 siRNA and polo-like kinase 1 (PLK1) siRNA to suppress the growth of B16F10 cells. Both siRNAs were incorporated into lipid-coated calcium phosphate (LCP) NPs. With a size of 40 nm and high loading capacity, siRNA-loaded LCP NPs could quickly release active agents in an acidic environment and downregulate PD-L1 and PLK1, resulting in anti-cancer efficacy [39].

#### 3.1.3. miRNAs

**Figure 4.**
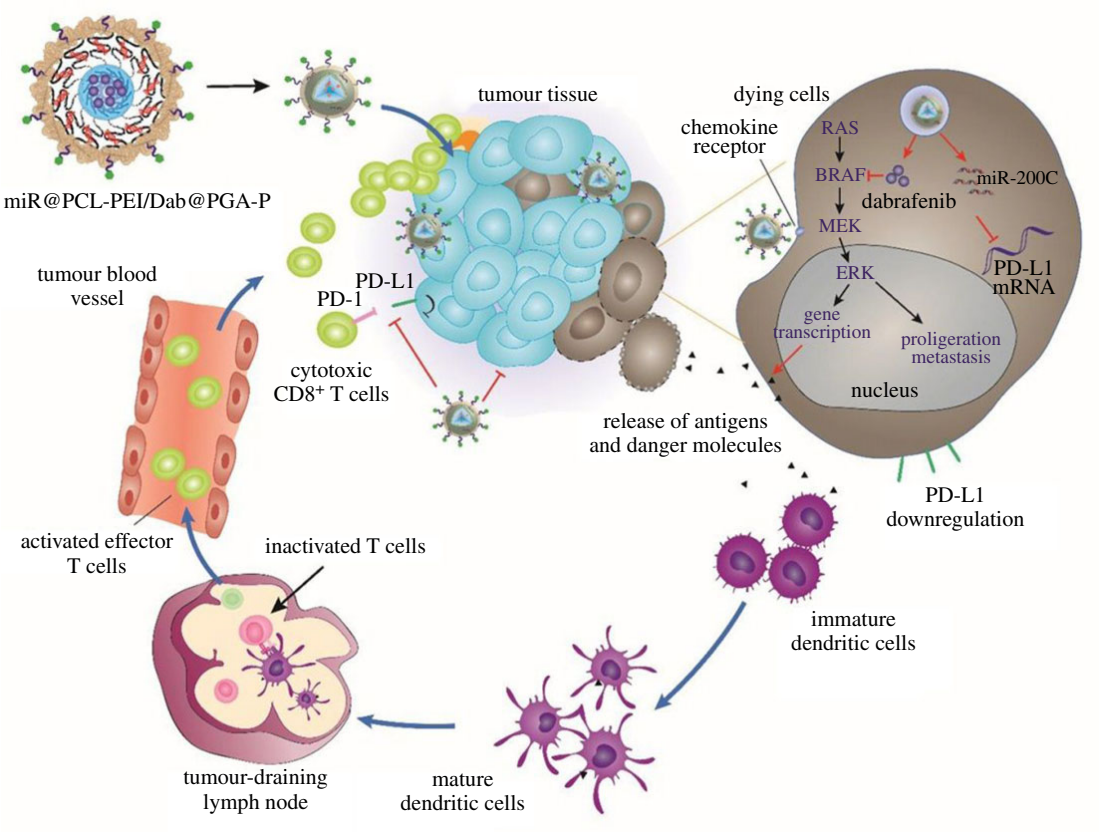
Development of miRNA-based nanosystems for immunotherapy. Manipulating immune system using NPs for an effective cancer treatment: combination of targeted therapy and checkpoint blockade miRNA. Reprinted with permission from [43].

miRNA is not as popular as siRNA, but some studies have reported promising results warranting the future development of this therapeutic agent. The earliest miRNA study investigated the combination of miR-200c and docetaxel, which were loaded into gelatinase-stimuli NPs that enhanced the cytotoxicity of docetaxel, possibly by decreasing β-tubulin III levels. This co-delivery approach accumulated and retained higher level of docetaxel at the tumour site, thereby suppressing *in vivo* tumour growth [40]. Subsequently, this research group used miR-200c to regulate PD-L1 expression and epithelial-mesenchymal transition, by which gastric cancer cells were vulnerable to radiotherapy and quickly eliminated. In this study, miR-200c was encapsulated into DSPE-PEG-NHS-based self-assembled micelles. Upon radiotherapy induction, miR-200c further inhibited the expression of PD-L1, demonstrating the potential application of miRNA in anti-cancer therapy [41]. Our group examined the efficacy of miR-200c via two different strategies. Phung *et al.* encapsulated doxorubicin and miR-200c into folate-receptor-targeted NPs. The targeting moiety enhanced the concentration of NPs and drugs to the tumour site, by which miR-200c suppressed PD-L1 expression. Concurrently, doxorubicin can kill cancer cells and induce immunogenic cell death, leading to increased dendritic cell maturation and tumour response of CD8^+^ T cells [42]. In a recent study, Nguyen *et al*. combined dabrafenib, a BRAF inhibitor and miR-200c into CXCR4-targeted NPs. The loaded PCL-PEI core was coated with poly-L-glutamic acid conjugated with a CXCR-4 antagonist peptide core that responded to pH and redox. This design could improve the delivery of therapeutic agents to tumours, increase effector T cells in tumours and suppress the expression PD-L1 and Tregs. Owing to their synergistic effect, these NPs are promising candidates for targeted immunotherapy (figure 4) [43].

#### 3.1.4. Peptides

Taleb *et al.* fabricated bifunctional therapeutic peptide-assembled NPs targeting the PD-1/PD-L1 immune checkpoint pathway. The NPs, at a size below 100 nm, contained peptide derivatives that could induce the normalization of the TME and anti-tumour immunogenic response. Upon *in vivo* treatment, the tumour volume of control group is increased from 0 to approximately 1400 mm^3^, whereas the tumour of the NP-treated group only increased from 0 to 400 mm^3^ suggesting the superior of peptide and doxorubicin-loaded nanosystem in managing tumour growth [44]. Previously, Cheng *et al.* used the same strategy but with different peptides to facilitate dual-targeted cancer immunotherapy. The onsite release of ^D^PPA-1 and NLG919 activated cytotoxic T lymphocytes, resulting in the effective inhibition of tumour growth [45].

A PD-1-targeting peptide, P–F4, was encapsulated into mPEG-PLA-based NPs, which could bind to PD-1 with an affinity of 0.119 μM. This loading not only increased CD8^+^ T cells and reduced Tregs in the TME, but also strongly inhibited tumour growth in a CT26-bearing mouse model. Compared with the doxorubicin-treated group, P-F4 NP-treated group showed similar efficacy but improved safety which was similar to that of the saline-treated group [46].

**Figure 5.**
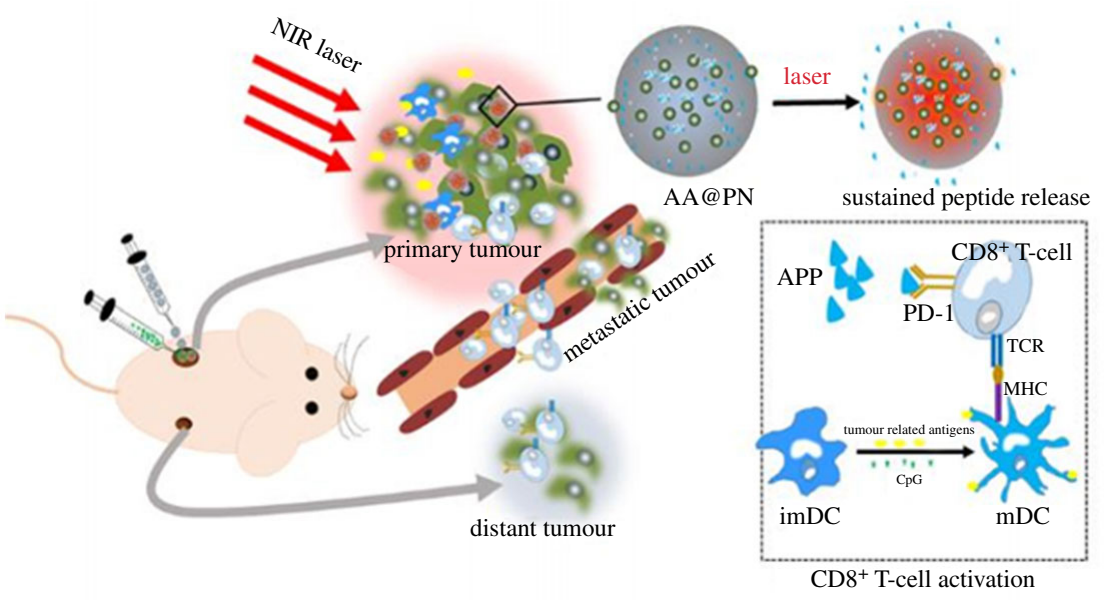
Development of peptide-based nanosystems for immunotherapy. Laser immunotherapy in combination with perdurable PD-1 blocking for the treatment of metastatic tumours. Reprinted with permission from [48]. Copyright 2021 American Chemical Society.

Using an anti-PD-1 peptide (sequence: (SNTSESF)_2_KFRVTQLAPKQIKE-NH_2_) as the PD-1 blocking agent, Luo *et al.* found that peptide-loaded PLGA NPs could prolong the release of peptides up to 40 days. The level of PD-1 in PBMCs and splenic lymphocytes isolated from CT26 tumour-bearing mice was significantly reduced, resulting in the inhibition of tumour growth [47]. This NP design was further incorporated into photothermal therapy in which gold nanoshells were co-encapsulated. This strategy could effectively eliminate primary tumours and suppress metastatic tumours (figure 5) [48].

Instead of targeting on T cells, Lee *et al.* developed a magnetic nanosystem containing the peptide AISLHPKAKIEES to manipulate NK cells. Magnetic NPs are easily attracted to liver tumour sites where the NK cells are activated, thereby enhancing the expression of nuclear factor kappa B (NF-κB), caspase 8 and caspase 3, which potentially benefit cancer treatment [49].

#### 3.1.5. Small molecules

**Figure 6.**
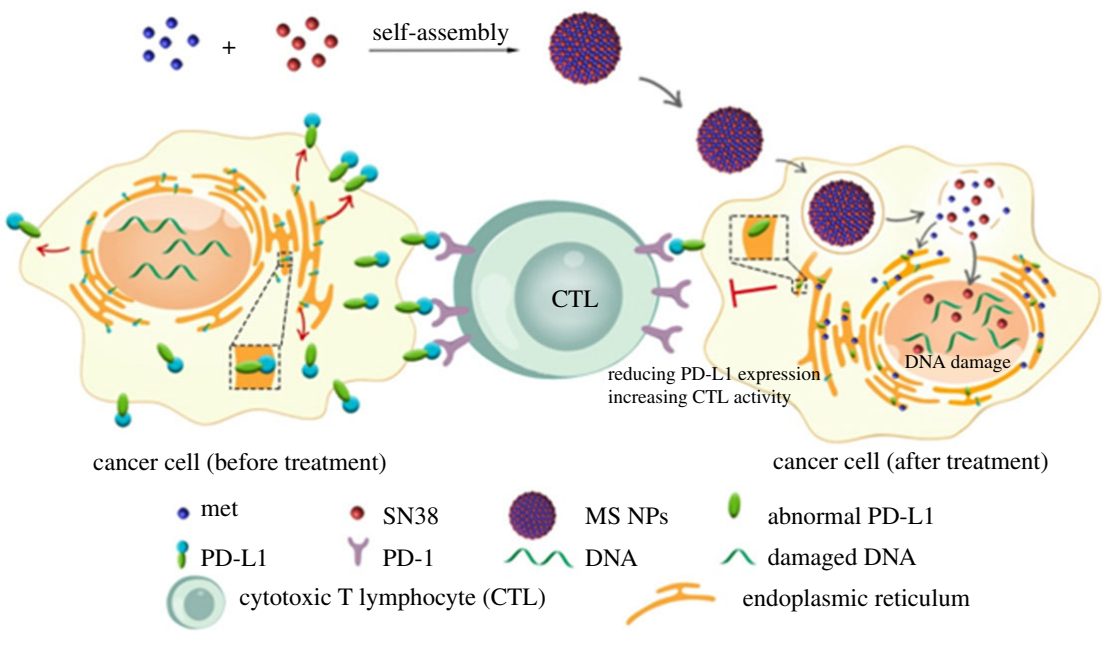
Development of small-molecule-based nanosystems for immunotherapy. Reducing PD-L1 expression with a self-assembled nanodrug: an alternative to PD-L1 antibody for enhanced chemo-immunotherapy [50] (published under a Creative Commons Attribution).

Antibodies, peptides or siRNAs are clinically proven as promising active agents. However, to meet the treatment requirements for a large number of populations, small molecules are still feasible and need to be further investigated. Recently, some small molecules have been encapsulated into nanosystems designed to transport newly synthesized and repurposed drugs. Yu *et al.* recruited a new immune checkpoint blockade agent, BMS-202, along with photothermal therapy. In this approach, BMS-202 was loaded into small-sized albumin NPs, which were then coated with a thermo- and fibrotic matrix-sensitive liposome. This nanosystem not only directly inhibited tumour growth, but also downregulated the PD-1/PD-L1 axis (figure 6) [51].

Among the repurposed drugs, metformin is the most promising candidate that has been reported in several studies. Wen *et al*. embedded metformin in a chitosan scaffold that was coated with a layer of manganese dioxide (MnO_2_). This drug delivery system enhanced the apoptosis of tumour cells via immunotherapeutic means both *in vivo* and *in vitro*. Therefore, it is suggested that chitosan-metformin@MnO_2_ particles could be applied for cancer immunotherapy as well as post-surgical wound healing [52]. In their work, Cai *et al.* combined the anti-cancer agent 7-ethyl-10-hydroxycamptothecin and metformin to downregulate PD-L1 expression. Intriguingly, both drugs could self-assemble to form NPs of 50 nm in size, which could attenuate the expression of PD-L1 in MDA-MB-231 cells. In combination, both drugs were retained longer in the blood circulation, resulting in a higher accumulation at tumour sites by twofold. Additionally, the use of both drugs also reduced the tumour size and prolonged the survival time up to the end of the study (30 days) compared with 16 days in the control group. Based on the *in vivo* immunofluorescence staining images, the expression of PD-L1 in the NP-treated groups was markedly decreased, whereas those of the effector T cells were increased (figure 6) [50].

In another recent study, metformin and IR775 were incorporated into a clinically usable liposome in which the nanosystem reduced PD-L1 expression, strengthened T-cell exhaustion and manipulated the tumour hypoxia by which bladder and colon cancers were suppressed both locally in the primary tumour and abscopally [53].

### 3.2. PD-1/PD-L1-targeting nanocarriers for anti-cancer therapies

Together with PD-1-targeting peptides, PD-L1 is a potential target. Sun *et al.* conjugated a PD-L1 recognizing peptide DPPA-1 with a tumour vasculature affinity peptide, which was then incorporated into the methoxy poly(-ethylene glycol)_3000_-poly(ε-caprolactone)_20000_ (MPEG-PCL) NPs. The paclitaxel-loaded NP was approximately 96 nm in size and could extend the survival time for tumour-bearing mice by 19.5 days and 47.5 days corresponding to PBS and nanosystem treatment groups, respectively [54]. In another study, an anti-PD-L1 peptide was conjugated with IR780 to form NPs. This nanosystem could accumulate effectively at the tumour site, and upon laser irradiation, the tumour was eradicated. In addition, the presence of peptides could alter the populations of CD8^+^ T cells, CD4^+^ T cells and Tregs, which lead to the restoration of TME homeostasis that favours the immune system to inhibit tumour growth. In this animal model, tumour growth was reduced along with an increase in effector T cells and immune-activated cytokines such as IL-6, IL-2, TNF-α and IFN-γ [55].

Prior to NP fabrication, Gurung *et al.* screened for PD-L1-binding peptides. Homology and structural analyses revealed the interaction between PD-L1 and either PD-L1 Pep-1 or PD-L1 Pep-2. By injecting PD-L1 Pep-1 and PD-L1 Pep-2, tumour growth inhibition and an increased CD8^+^/FoxP3+ ratio in mice were observed, whereas upon co-delivery of peptides and doxorubicin using the liposomes, the tumour was completely removed, and the proportion of CD8^+^ T cells was significantly increased [56].

Hasanpoor *et al.* used a PD-L1 binding peptide (TYLCGAISLAPKAQIKASL) to decorate HSA-curcumin NPs. With a size of 246 nm, peptide-HSA/Cur NPs were effectively internalized into PD-L1-expressing breast cancer cells. Although this nanosystem enhanced cancer cell death, a controlled *in vivo* study should be conducted to validate the results of this approach [57].

Zhu *et al.* demonstrated potential chemo-immunotherapy efficacy by conjugating a PD-L1-binding peptide with two hydrophobic stearyl chains via a pH-sensitive linker. This novel material forms a complex with doxorubicin. Within the acidic tumour microenvironment, the NPs are disintegrated to release doxorubicin, hence, inducing chemotherapy and immunotherapy simultaneously. This combination approach significantly inhibited CT26 tumours and induced an immune response both *in vitro* and *in vivo* [58].

### 3.3. PD-1/PD-L1-targeting NPs for cancer theranostics

In this section, the PD-1/PD-L1-targeting NPs are reviewed as a tool for both diagnosis and treatment named theranostics. Du *et al.* fabricated PD-L1-nanohybrid liposomal cerasome NPs that were decorated with PD-L1 antibodies for tumour targeting. In addition, this nanosystem also carried paclitaxel as a chemo-agent, IRDye800CW as a near-infrared fluorescent (NIRF) and Gd-DOTA as a MRI contrast agent. This paclitaxel-loaded nanocarrier showed potential efficacy in eliminating cancer cells, both *in vitro* and *in vivo*. Additionally, these IRDye800CW and Gd-DOTA-bearing NPs offer a strong assessment for the *in vivo* NIRF and MRI imaging of 4T1 breast tumours and CT26 colon tumours. The inclusion of PD-L1 antibody on this nanosystem could enhance the signal intensity [59]. In another study, human PD-L1 peptides were conjugated into gold nanoprisms for multi-functional purposes, including photothermal/photodynamic therapy. Moreover, the encapsulation of chlorin e6 into this system offered an opportunity to probe HCC827 tumours using fluorescence imaging and photoacoustic imaging [60].

### 3.4. PD-1/PD-L1 blocking and combination perspectives

PD-1/PD-L1 blocking agents in free form or encapsulated form are usually used in combination with other therapies to yield synergistic effects. Thus far, phototherapies have been the most frequently applied, including photothermal and photodynamic therapies. To enhance treatment efficacy, the application of NPs is enhanced using a peptide/antibody targeting the PD-1/PD-L1 axis, and the photo-absorbed agents are highly diverse in design and formulation. For example, Au@Pt NPs could generate NIR irradiation-induced heat to kill cancer cells and activate T cells using anti-PD-L1 peptide [61]. In devising new treatment modalities for lung cancer, Liu *et al.* created gold nanoprisms for photothermal therapy in combination with human PD-L1 siRNA. Laser exposure could generate heat up to 60°C which could strengthen the cytotoxic effect of the nanosystem [26]. In another study, PLGA-PEG NPs loaded with iron oxide and perfluoropentane were used to treat melanoma. Upon laser irradiation, the temperature at the tumour site could be increased by 25°C to trigger the release of encapsulated anti-PD-1, leading to increased CD8^+^ T-cell infiltration and immunotherapy efficacy [20]. The programmed release of anti-PD-1 peptide was also applied when anti-PD-1 peptide and hollow gold nanoshells were co-loaded into PLGA NPs. At about 55°C after NIR trigger, the release of anti-PD-1 peptide was significantly increased, resulting in a synergistic cytotoxic effect on the tumour [47].

Similar to photothermal therapy, photodynamic therapy could be used to induce the generation of ROS upon laser exposure, which has shown remarkable treatment efficacy. Wang *et al.* synthesized NPs containing IR780 and an anti-PD-L1 peptide. This self-assembled nanosystem blocked PD-L1 and demonstrated improved anti-tumour efficacy in combination with photodynamic immunotherapy [55]. This strategy employed a couple of combinations such as anti-PD-1 and Ce6 [22], BMS-202 a small-molecule inhibitor of the PD-1/PD-L1 and Ce6 [62], the photosensitizer BDP-I-N and anti-PD-L1 [63], the photosensitizer mTHPC, and anti-PD-L1 [64] in nanocarriers.

The combination therapy involving radiotherapy and PD-1/PD-L1 blockade has been rarely reported. Thus far, only Qian *et al.* took advantage of this strategy while using miRNA-200c as the blocking agent [41]. The wide adoption of radiotherapy and its frequency of use might further boost this combination approach in the future.

In addition to surgical treatment, chemotherapy has been the most adopted cancer treatment approach; hence, it is logical to combine chemotherapy and immunotherapy. Liang *et al.* recruited 7-ethyl-10-hydroxycamptothecin and the STING agonist DMXAA (5,6-dimethylxanthenone-4-acetic acid) into triblock copolymer NPs, named PS3D1@DMXAA. This nanosystem synergized with anti-PD-1 therapy to convert ‘cold’ tumours into ‘hot’ tumours and, thereby, induced an anti-tumour effect in B16.F10 tumour-bearing mice [65]. In addition, using anti-PD-L1 as targeting purposes, Xu *et al.* encapsulated docetaxel into polyethylene glycol-poly(ε-caprolactone) NPs to treat gastric cancer. Although only *in vitro* assays were conducted, this approach was effective at enhancing the cellular uptake of NPs [66]. In a treatment model using SCC VII-bearing mice, Lan *et al.* first loaded cisplatin into pH-responsive tumour-targeted lipid NPs and then decorated them with anti-PD-1. This nanosystem was embedded into micro-needles, which could elicit an immune response to the tumour. Moreover, the number of CD8^+^ and CD4^+^ T cells was increased, whereas Treg cells were reduced [19]. In another study, doxorubicin and siPD-L1 were incorporated into poly-L-lysine–lipoic acid reduction-sensitive and tumour extracellular pH-stimulated NPs. As expected, there was a reduction in PD-L1 level, resulted in a synergistic effect in tumour growth inhibition following the induction of doxorubicin and upregulation of CD8^+^ T cells [32].

**Figure 7.**
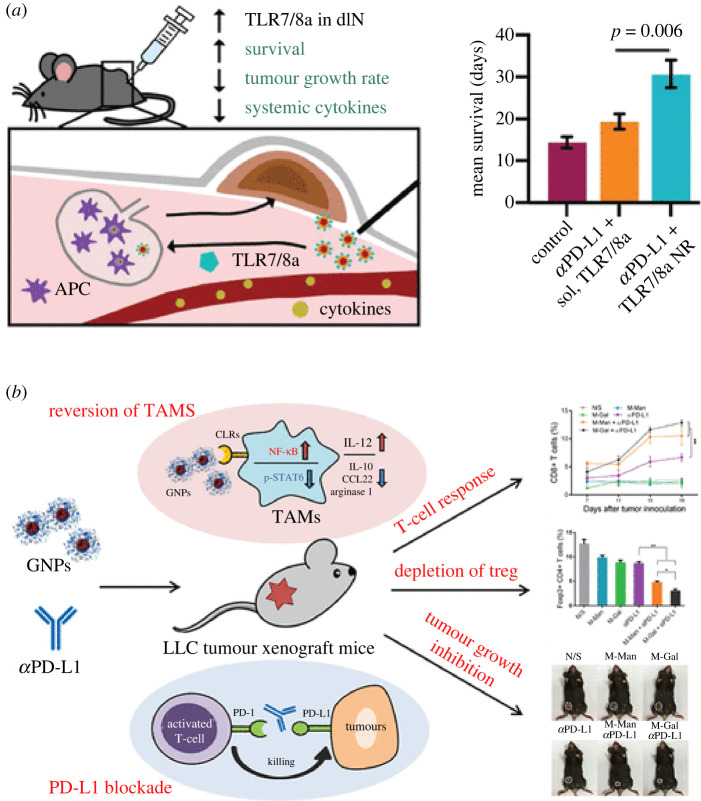
Combination approaches targeting PD-1/PD-L1 for anti-cancer immunotherapies. (*a*) NPs presenting potent TLR7/8 agonists enhance anti-PD-L1 immunotherapy in cancer treatment [68] (published under a Creative Commons Attribution). (*b*) Glycocalyx-mimicking NPs improve anti-pd-l1 cancer immunotherapy through reversion of tumour-associated macrophages. Reprinted with permission from [69].

There is a growing body of evidence on the application of immunotherapy in real life. Blockade of the PD-1/PD-L1 axis could be strengthened by the addition of other immune targets. Toll-like receptors expressed mostly on antigen-presenting cells, especially DCs, to probe the pathogen could offer an approach for combination therapy in which the active agents activate the immune system via PD-1/PD-L1 and TLRs in parallel [67]. By loading potent TLR7/8a moieties on the surface of poly(ethylene glycol)–poly(lactic acid) (PEG–PLA) NPs conjugated with anti-PD-L1, Smith *et al.* extended the survival period of mice from 13 days to more than 30 days. More importantly, this combination therapy significantly reduced the size of targeted tumour (figure 7*a*) [68]. In another combination set, anti-PD-1 was combined with the Toll-like receptor 9 agonist, CpG in the CuS NP system, which enhanced the efficacy of ovarian cancer treatment in a poorly immunogenic syngeneic ID8-ip1-Luc ovarian tumour model [21].

To another target, tumour-associated macrophages, the anti-PD-L1 was used along with the amphiphilic diblock copolymers poly(mannopyranoside/galactopyranoside methacrylate)-block-polystyrene NPs that upregulated the immunostimulatory IL-12 and downregulated immunosuppressive IL-10, arginase 1 and CCL22. This immuno-alteration affected the tumour-associated macrophages and manipulated the immunosuppressive TME, leading to treatment efficacy of this cancer immunotherapy (figure 7*b*) [69].

## 4. Future perspectives and conclusion

The robust development of checkpoint inhibitors targeting PD-1/PD-L1 paves the way for more effective tumour immunotherapy. In the clinical setting, only some patients respond to the therapy using the FDA-approved antibodies, such as nivolumab and pembrolizumab, hence, this necessitates the identification of novel predictive biomarkers. A minimum PD-L1 expression level of 50% was associated with clinical benefit using PD-1/PD-L1-targeting therapy [67], although this has been reported as the optimal threshold [70]. Therefore, to ensure the efficacy of this approach, the accurate diagnosis of tumour is critical, and therefore, by leveraging on the development of imaging technology, NPs can provide treatment feasibility.

An individual approach is not adequate to manage the complexity of cancer; hence, the combined and targeted therapy should be investigated further. Chemotherapy and radiotherapy are known for their contributions to fighting cancer, whereas phototherapy and additional immunotherapies offer a new horizon for combination therapy. Although phototherapy could be modulated via external stimulation, immunotherapy offers the advantage of a sustainable effect with fewer adverse events. Moreover, nanotechnology offers precision and reliability in delivering the active agent cargo with minimum leaking to the correct destination and at the right concentration.

The ultimate aim of cancer research and development is translating basic research to clinical applicability, which requires great effort in many aspects. Among the various reported nanosystem models, liposomes or lipid NPs are the most promising candidates fabricated based on high-quality formulations using less complicated synthesis procedures. At the present, some commonly used liposomes demonstrated the capability to carry the targeting moieties and respond to environmental conditions, including pH, temperature and enzyme [71]. However, nanomedicines with the passive targeting were demonstrated as more promising in preclinical studies but not that much in the clinical trials. This fact requires the deeper involvement of targeting strategy to enhance the accumulation of nanomedicines to the tumour then the efficacy could go along. The recent development in the field of material science also opened up a new paradigm of ‘*design as wished*’ for nanotechnology application in cancer treatment.

In addition to regulation concerns, the cost of therapy and financial burden among patients choosing this novel therapy should be taken into consideration. Therefore, by leveraging on nanotechnology, substantial investment on the optimization of small-molecule synthesis should be considered a noble endeavour that could strengthen the efficacy of this novel therapy.

Taken together, nanotechnology has improved the efficacy of PD-1/PD-L1-targeting immunotherapy for cancer treatment. PD-1/PD-L1 is an important immune checkpoint that plays a critical role in cancer management. Blockade of this target could enhance the infiltration of effector T cells into tumours and improve the sensitivity of cancer cells to immunotherapy, resulting in tumour suppression. The integration of nanotechnology offers increased precision in the target specificity, enhanced bioavailability of therapeutic agents, and diagnostic ability. A variety of active agents, including antibodies, peptides, siRNAs and small molecules, have been successfully incorporated into organic/inorganic nanosystems, offering great hope for the cure of cancer in the future.

## Data Availability

This article has no additional data.
